# Automated high-throughput high-content autophagy and mitophagy analysis platform

**DOI:** 10.1038/s41598-019-45917-2

**Published:** 2019-07-01

**Authors:** Jonathan Arias-Fuenzalida, Javier Jarazo, Jonas Walter, Gemma Gomez-Giro, Julia I. Forster, Rejko Krueger, Paul M. A. Antony, Jens C. Schwamborn

**Affiliations:** 1 Luxembourg Centre for Systems Biomedicine (LCSB), University of Luxembourg, 7 avenue des Hauts-Fourneaux, Esch-sur-Alzette, Luxembourg; 2 Laboratory of Developmental and Cellular Biology, Esch-sur-Alzette, Luxembourg; 3 Laboratory of Clinical & Experimental Neuroscience, Esch-sur-Alzette, Luxembourg; 4 Max Planck Institute for Molecular Biomedicine, Laboratory of Cell and Developmental Biology, Roentgenstrasse 20, Muenster, Germany; 5 Graduate School of Biostudies, Kyoto University, Kyoto, 606-8501 Japan

**Keywords:** Mitophagy, Induced pluripotent stem cells

## Abstract

Autophagic processes play a central role in cellular homeostasis. In pathological conditions, the flow of autophagy can be affected at multiple and distinct steps of the pathway. Current analyses tools do not deliver the required detail for dissecting pathway intermediates. The development of new tools to analyze autophagic processes qualitatively and quantitatively in a more straightforward manner is required. Defining all autophagy pathway intermediates in a high-throughput manner is technologically challenging and has not been addressed yet. Here, we overcome those requirements and limitations by the developed of stable autophagy and mitophagy reporter-iPSC and the establishment of a novel high-throughput phenotyping platform utilizing automated high-content image analysis to assess autophagy and mitophagy pathway intermediates.

## Introduction

Autophagy and mitophagy play central roles in normal development and disease^1,2^. Increasing interest and research in the field point out the need of pathway reconstitution tools and reliable quantification methods of autophagy and mitophagy^3,4^. Analyses of these processes have so far been conducted via low-throughput and semi-quantitative methods such as transmission electron microscopy, transient transfections, and western blot^5^. Current methodologies to assess autophagy and mitophagy impairments miss the multiple structural intermediates of autophagy and mitophagy pathways. The implementation of technologies for identifying the successive developing structures in the autophagy and mitophagy pathway (staging) is necessary to dissect the impaired steps of the pathways. Such technologies enable stratifying and categorizing pathologies that affect these crucial homeostasis pathways, and reveal new pharmaceutical targets^6^. The advent of genome editing tools has accelerated the development of genetically encoded reporters in human induced pluripotent stem (iPS) cells^7,8^. Additionally, the advancement of pH-responsive fluorescent proteins allows evaluating intracellular pH and interrogating specific subcellular compartments^9,10^. Here, we harnessed said advancements to overcome the outlined limitations in the autophagy field, by applying them in conjunction with automated imaging and automated analysis pipelines. We utilized the Rosella-system, a well-established fluorescent protein-based sensor (DsRed and pHluorin), for monitoring lysosome-converging pathways and cargo. The pHluorin (modified GFP) signal is quenched in acidic environments like the autolysosome lumen. At the same time its companion DsRed remains fluorescent. Thus, events presenting only DsRed ultimately indicate fusion with lysosomes. Since the use of the Rosella system in yeast^11^, the pH sensitivity of pHluorin was further optimized^12^ and lately applied in human cells^10^. Our approach relies on the stable expression of the Rosella reporter, which is integrated into the AAVS1 locus^13^ using TALE nucleases guided targeting. It represents an additional step in the technical improvement of the Rosella system and enables a high level of detail in the analysis.

After validation, we utilized the stable reporter cells to acquire and quantify the respective pathway intermediates in our high-throughput phenotyping platform employing automated image analysis. Automated high-throughput high-content imaging and analysis approaches have multiple advantages over conventional methods. Automated tools allow applying classification algorithms to specimens in an unbiased manner, and provide high statistical power. Importantly, those tools provide an insight to the flow of these pathways that would otherwise be missed in population-based analyses. Here, we present such automated tools to assess autophagy and mitophagy intermediates.

## Results

### Reconstruction of autophagy and mitophagy pathway intermediates

**Figure 1:**
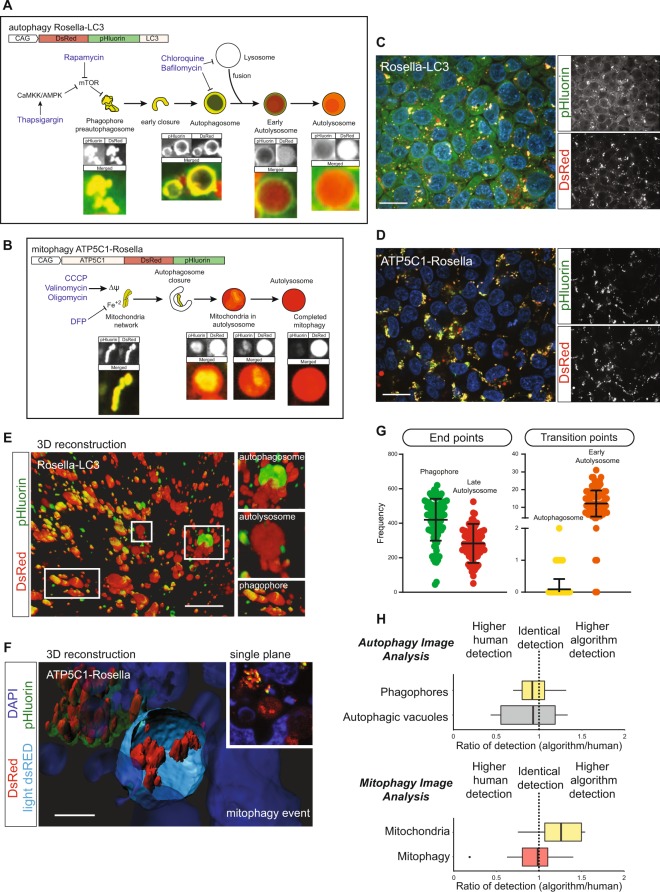
Genetically encoded Rosella-LC3 and ATP5C1-Rosella systems allow monitoring of the complete autophagy and mitophagy pathway. (**A**) Structure of the Rosella autophagy reporter system. It is possible to identify phagophores, autophagosomes, and autolysosomes. Small molecule modulators of autophagy can interrogate the autophagy responsiveness. (**B**) Structure of the Rosella mitophagy reporter system. It is possible to determine mitochondrial network structure and mitophagy events. Small molecule mitochondria stressors can test the mitophagy capacity. Contrast of displayed images in (**A**) and (**B**) was adjusted by saturating the bottom and the top 1% of all the pixels values. (**C**) Representative field for Rosella-LC3 line. The pHluorin and DsRed channels are shown separately. Scale bar, 10 µm. (**D**) Representative field for ATP5C1-Rosella line. The pHluorin and DsRed channels are shown separately. Scale bar, 10 µm. (**E**) 3D reconstruction based on the Rosella-LC3 line. The insets show DsRed^pos^pHluorin^pos^ autophagosome structures, DsRed^pos^pHluorin^neg^ autolysosome structures, and DsRed^pos^pHluorin^pos^ phagophores. Scale bar, 10 µm. (**F**) 3D reconstruction based on the ATP5C1-Rosella line. An autolysosome structure with an ongoing mitophagy event is shown. The autolysosome appears with an equatorial cross section and the light DsRed volume is represented in cyan. The residual mitochondria inside the autolysosome are pHluorin^neg^, maintaining the pH-resistant DsRed signal. A phagophore DsRed^pos^pHluorin^pos^ cluster is located in the upper left-hand corner. In the inset, a single plane overlay is shown. The event was observed upon addition of 5 µM valinomycin. Scale bar, 4 µm. (**G**) Absolute quantification of phagophores, autophagosomes, early autolysosomes and late autolysosomes per field. At seeding density of as 600k cells/cm^2^, a range of 40–60 cells per field was obtained. **(H)** Level of detection of the algorithm compared to manually segmented images (n = 10). Events were manually segmented by establishing a region of interest (ROI) around each event detected and compared to the events detected by the automated image analysis. No statistical difference (t-test) was observed between the manual and algorithm segmentation for the different classes.

Making use of genetically encoded pH sensors and genome editing tools, we have engineered an iPSC line to monitor autophagy and mitophagy. The autophagy sensor Rosella-LC3 allows the identification of pre-autophagosomal structures such as phagophores, and transient structures such as isolation membranes and autophagosomes (Fig. 1A,C,E). Following autophagosome fusion with lysosomes, the internal membrane bound LC3 is degraded, giving rise to early and then late autolysosomes (Fig. 1A,C,E) that converge to lysosomes and re-enter the autophagy cycle^14,15^. The mitochondrial sensor ATP5C1-Rosella allows the quantification of the rate of mitophagy events (Fig. 1B,D,F) as accounted by acidic DsRed^pos^pHluorin^neg^ vesicles derived from degraded mitochondria. Using pattern recognition algorithms, we identified and categorized the subcellular structures observed during autophagy (Fig. 1G). Autophagosomes and early autolysosomes are transient intermediates with low frequency levels, compared to the frequency of events at the extremes of the pathway: phagophores and late autolysosomes (Fig. 1G). Autophagic-vacuoles comprise all autophagosomes and autolysosomes in a cell^5^. The ratio between phagophores and autophagic-vacuoles relates to a chemical reaction rate-constant. This metric accounts for the autophagy rate-constant intrinsic to a cell line in defined conditions. In the here investigated example, the autophagy rate was 0.41 under basal conditions.

Validation of the detection capacity of the algorithm was evaluated by comparing the results obtained from manual segmentation of events and those coming from the autophagy and mitophagy image analysis (Fig. 1H). No statistical difference was observed between the detection done by the algorithm and that from manual segmentation for all the categories (t-test between manual and algorithm segmentation; Phagophore: *p* = 0.25, Late Autolysosome: *p* = 0.70, Mitochondria: *p* = 0.11, Mitophagy: *p* = 0.55), although a higher performance for detecting mitochondria events was observed.

### Autophagy image analysis decision tree

**Figure 2:**
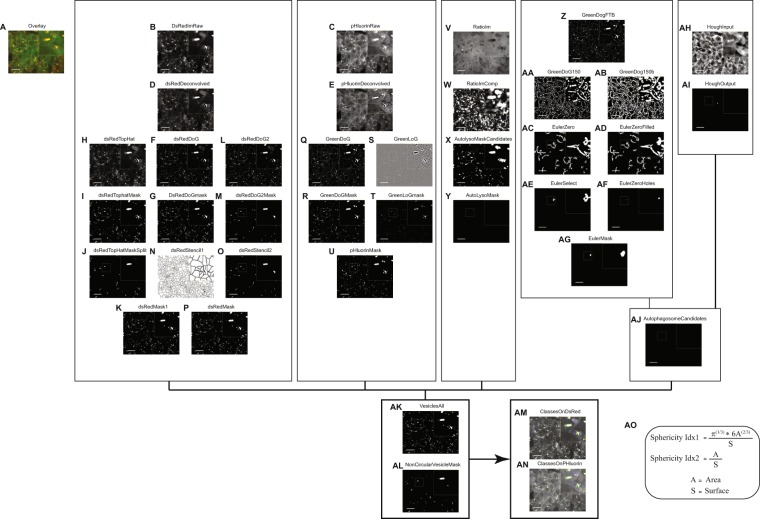
Image analysis workflow for autophagy Rosella-LC3 reporter line. (**A**) Overlay of raw DsRed and pHluorin channels. (**B**) Raw image for DsRed, dsRedImRaw. (**C**) Raw image for pHluorin, pHluorinImRaw channel. (**D**) Deconvolved image of DsRed channel, dsRedDeconvolved. (**E**) Deconvolved image of pHluorin channel, pHluorinDeconvolved. (**F**) Application of a difference of Gaussian filter, dsRedDoG, and its respective mask, dsRedDoGmask (**G**). (**H**) Top-hat filtering to improve the detection of red vesicles, dsRedTopHat, and its respective mask, dsRedTopHatMask (**I**). (**J**) Refinement of (**I**) by substitution with (**G**), dsRedTopHatMaskSplit. (**K**) Combination of (**G**,**J**), dsRedMask1. (**L**) To confirm the detection of red vesicles, a second difference of Gaussian was applied, dsRedDoG2, and its mask was created, dsRedDoG2Mask (**M**). (**N**) Using (**K**) as a seed, a watershed function was applied, dsRedStencil1. (**O**) Elementwise multiplication of (**N**) with (**M**), dsRedStencil2. (**P**) Pool of pixels in (**K**,**O**), dsRedMask. (**Q**) Application of a difference of Gaussian filter, GreenDoG, and its respective mask, GreenDoGMask (**R**). (**S**) Application of a Laplacian of Gaussian filter, GreenLoG, and its respective mask, GreenLoGmask (**T**). (**U**) Combination of (**T**,**R**), pHluorinMask. (**V**) Elementwise division between blurred (**C**) and blurred (**B**), RatioIm, and calculation of its complement, RatioImComp (**W**). (**X**) Top-hat filtering and its respective mask, AutoLysoMaskCandidates. (**Y**) Validation of (**X**) based on neighborhood pixel intensity, AutoLysoMask. (**Z**) Filtering of (**Q**) with a Butterworth high pass filter, GreenDoGFTB, and its respective mask, GreenDoG150 (**AA**). (**AB**) Opening of the (**AA**) mask, GreenDoG150b. (**AC**) Euler numbers used to detect holes. Elements with only one hole were kept, EulerZero. (**AD**) Pre-processing of (**AC**) by filling holes, EulerZeroFilled. (**AE**) Selection of (**AD**) based on the proportions between object and hole size, EulerSelect. (**AF**) EulerZeroHoles, and its respective mask, EulerMask (**AG**). (**AH**) Hough transforms for circle detection on (**C**), HoughInput. (**AI**) Validation of (**AF**) based on the pixel values of (**B**,**C**), HoughOutput. (**AJ**) Combination of (**AG**) and (**AI**), AutophagosomeCandidates. (**AK**) Mask based on the mean intensity in (**B**) of all connected components, VesiclesAll. (**AL**) The remaining connected components of (**AK**) were evaluated on eccentricity, NonCircularVesicleMask. (**AM**) All vesicles mapped on DsRed channel. (**AN**) All vesicles mapped in pHluorin channel. Contrast of displayed images was adjusted by saturating the bottom and the top 1% of all the pixels values. Diameters shown in the results section correspond to the major axis length of the respective vesicles. Scale bars indicate 20 µm and 3x zoomed insets are highlighted with yellow boxes. **(AO)** Formula for calculating the sphericity indices used in the analysis.

As shown in (Fig. 2), an automated computational image analysis workflow for the resulting multichannel 3D images was implemented. A detailed explanation of the decision tree can be seen in the methods section, and it is briefly summarized here. The workflow was based on imaging of living cells and we had to account for some technical limitations outlined below. Segmentation of the cells based on the fluorescence signals of Rosella-LC3 or ATP5C1-Rosella in combination with nuclear Hoechst staining was insufficient to achieve reliable segmentation of single cells. To account for that problem we decided to apply field-imaging on high-density cultures assuming a similar number of cells per imaging field, which was shown to be a reliable approach^16^. We accounted for the reduction of statistical power by elevating the number of fields acquired per well. Images were deconvolved and an estimation of the point spread function generated with the PSFGenerator tool^17^. After deconvolution of the DsRed channel, a difference of Gaussians, subtracting a convoluted foreground image with a background one, was computed in order to highlight DsRed^pos^ vesicles via spatial bandpass filtering. To further improve the sensitivity of detection for DsRed^pos^ vesicles, this approach was complemented with a top-hat filtering approach. Vesicles coming from this mask were further split using them as seeds for a Euclidean distance transform and a watershed function. To confirm the detection of DsRed^pos^ vesicles, a second difference of Gaussians was computed with different background and foreground settings. The final DsRed^pos^ mask was computed via Boolean operation by pooling all pixels which were detected so far. For adding more sensitivity for the detection of non-acidic vesicles, segmentation steps based on the deconvolved pHluorin channel were added. A difference of Gaussians was computed to segment larger vesicles, and a Laplacian of Gaussian was used to detect the edges of the vesicles.

Ratio images were computed by elementwise division between the blurred pHluorin image and the blurred DsRed image to detect autolysosomes. To detect autophagosomes based on their hollow vesicle property, an algorithm combining Fourier transform, Euler filtering, and Hough transform was implemented.

The classification of the segmented vesicles was designed to identify four vesicle types, namely phagophores, autophagosomes, early autolysosomes, and late autolysosomes, via a progressive exclusion algorithm. The classification decision is based on the masks of connected components calculated, and their intensities in the respective channels.

### Human iPS cells present active and dynamic autophagy and mitophagy

We observed remarkable activity of the autophagy pathway in human iPS cells (Videos S1–4). Indeed, autophagy has previously been associated with the maintenance of pluripotency and resistance to senescence in stem cells and proved essential for pre-implantation development^1^. Furthermore, we observed autolysosome vesicles interacting with the surface of the mitochondrial network and abundant mitophagy events (Videos S5–7). This is in agreement with reported mechanisms^18,19^ in this cell type^20,21^.

### Responsiveness to a panel of autophagy and mitophagy modulators

**Figure 3:**
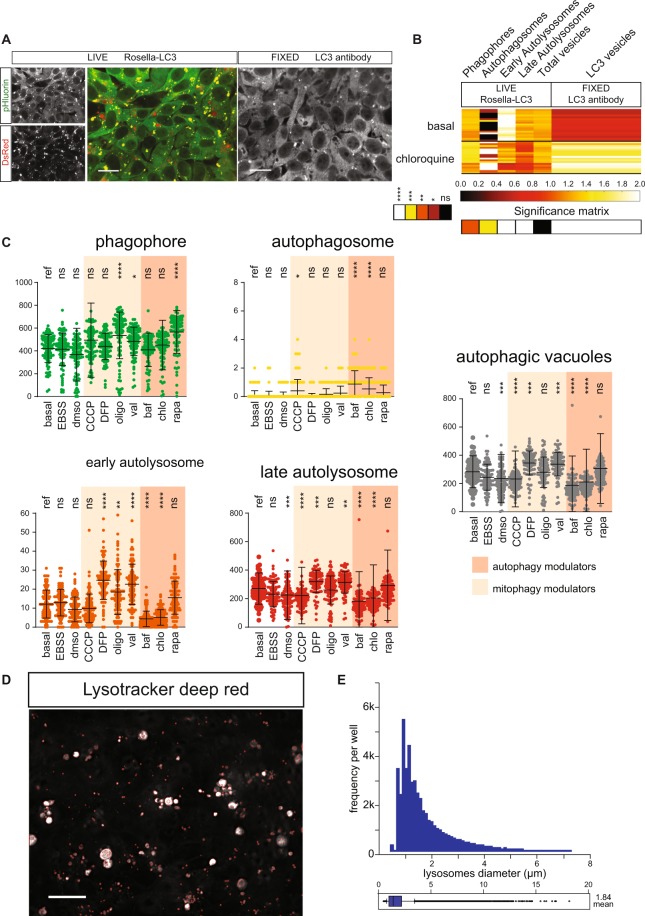
Quantification of autophagy structures before and after addition of mitophagy or autophagy modulators. (**A**) Representative fields of live Rosella-LC3 and fixed LC3 antibody. Scale bar, 20 µm. Contrast of displayed images was adjusted by saturating the bottom and the top 1% of all the pixels values. (**B**) Heatmap of scaled (0.0–2.0) category-mean normalized absolute frequency per well for autophagic structures for live Rosella and fixed LC3 antibody Rosella-LC3. Each line in the heatmap represents the averaged amount of vesicles observed of a well. Significance matrix of Kruskal-Wallis and Dunn’s multiple comparison test is shown below. (**C**) Autophagic-vacuoles are quantified as the sum of autophagosomes, early autolysosomes, and late autolysosomes. Dunnett’s multiple comparison of means was performed for all conditions with respect to the basal reference (ref). Standard deviations are shown. Each dot represents the sum of objects in one imaged field. Data represent three independent replicates. (**D**) Representative lysotracker staining. Lysosome mask on red perimeter. Contrast of the image was adjusted by saturating the bottom and the top 1% of all the pixels values. Scale bars indicate 20 µm. (**E**) Lysosome frequency and diameter in basal conditions. Figure significance levels are *p < 0.05, **p < 0.01, ***p < 0.001, and ****p < 0.0001.

Our 3D analysis algorithm enables absolute quantification of single autophagy events of cells in monolayer cultures. In order to validate this approach with traditional immunostaining, we performed high-content quantification of LC3-positive structures (Fig. 3A,B). Each line in the heatmap (Fig. 3B) represents the averaged amount of vesicles observed of a well. The resolution of conventional LC3-antibody staining lacks the ability to resolve the stage-specific structures in the autophagy pathway. In contrast to LC3-antibody assays, the reporter lines used in the present work enable classification based on acidic content. LC3-antibody quantification, is limited to total vesicle count, lacking pathway intermediates (Fig. 3B). Using chloroquine, a known inhibitor of the autophagy pathway flow at the autophagosome level^22^, a significant increase (Kruskal-Wallis and Dunn’s multiple comparison test) in LC3 positive vesicles was observed in both cases, however the cells presenting the Rosella construct depict that this rise is due to an increase in phagophores and autophagosomes. This demonstrates that the blockage induced by chloroquine generates an accumulation of vesicles at the early stages of autophagy.

In order to validate our combined systems of stable reporters and new automated quantification tools, we evaluated the responsiveness to autophagic and mitophagic stress and modulation. We used validated small molecule perturbations for this approach. Upon addition of the H^+^-ATPase inhibitor bafilomycin, acidification of lysosomes was impaired. In agreement with the expected blockage of trafficking^5^, we observed a significant increase (ANOVA and Dunnett’s multiple comparison) in the level of autophagosomes, and a decreased abundance in autophagic-vacuoles (Fig. 3C). Likewise, chloroquine led to the accumulation of autophagosomes and a decrease in autophagic-vacuoles (Fig. 3C).

It is known that mTOR complex 1 inhibits autophagy by its interaction with ULK1, resulting in reduced formation of the phagophore complex^23^. Rapamycin, an inhibitor of mTOR, allows quantification of the direct extent of autophagy controlled by this pathway. Addition of rapamycin increased phagophore levels as expected (Fig. 3C). In order to evaluate the contribution of mitophagy to general autophagy, we used a panel of established mitophagy inducers^10^. Oligomycin, valinomycin, and CCCP are modulators of the mitochondrial membrane potential that induce mitochondrial stress and mitophagy. Only oligomycin and valinomycin induced an increase in phagophore levels (Fig. 3C). Stress induced by DFP and valinomycin induced an increase in autophagic vacuoles (Fig. 3C). Known responses by mild stressors might be important for inducing an increase in Z’ values when doing assay development for drug screening purposes.

To determine the acidification capacity of autophagic-vacuoles, we complemented the autophagy and mitophagy reporters with a lysosomal dye (Fig. 3D,E) and developed a lysosome recognition algorithm (Fig. S1). This analysis completes the phenotyping of the lysosmal-autophagosomal axis and allows discarding impairments in the flow of the autophagy pathway that are due to a decreased acidification capacity of autolysosomes.

### Assessment of mitochondria network volume and mitophagic vesicles volume

**Figure 4:**
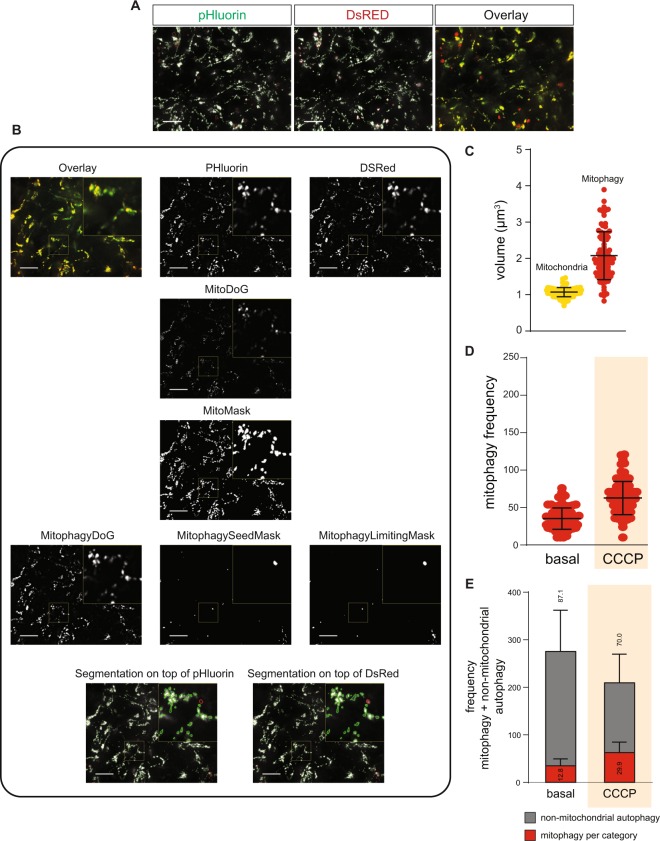
Evaluation of mitochondria network and mitophagic-vacuoles. (**A**) Representative images of ATP5C1-Rosella reporter. Mitochondria network mask on green perimeter and mitophagic-vacuole mask on red perimeter. Scale bars indicate 20 µm. (**B**) Image analysis workflow for mitophagy with ATP5C1-Rosella reporter line. Scale bars indicate 20 µm and 3x zoomed insets are highlighted with yellow boxes. Contrast of displayed images was adjusted by saturating the bottom and the top 1% of all the pixels values. (**C**) Mitochondrial and mitophagy volumes in basal conditions. (**D**) Mitophagy frequencies in basal condition and after mitochondrial stress induction. (**E**) Evaluation of mitophagy levels and distribution of autophagy resources upon mitochondrial stress. Percentages for mitophagy and non-mitochondrial autophagy are indicated.

Next, we quantified the mitochondrial network and mitophagic-vacuoles volume with the aid of pattern recognition algorithms (Fig. 4A–D). For a detailed explanation of the workflow, please refer to the methods section. The segmentation of mitochondria was achieved by a DoG of convoluted foreground and background images. The mitophagy events were detected via a combination of green to red fluorescence ratio analysis and morphological filtering based on difference of Gaussians thresholding was used. Resulting segmented mitochondria and mitophagic vacuoles are shown for DsRed and pHluorin (Fig. 4B, lower panel).

The disparity between the volumes of a mitochondrion and mitophagic vacuoles during the process of macroautophagy (Fig. 4C) could be explained by different scenarios: several mitochondria prone to be degraded could be engulfed by the same vesicle, or addition of several lysosomes to the already formed autolysosome increases the vesicle size, or fusion of late autolysosome vesicles might occur. Upon addition of the mitochondrial stressor CCCP, the algorithm was able to detect an increase in the frequency of mitophagic events (Fig. 4D).

Afterwards, we dissected the balance between mitophagy and general autophagy. The Rosella-LC3 sensor quantified the combined effect of non-mitochondrial autophagy and mitophagy. To model non-mitochondrial autophagy and to infer how phagocytic resources are distributed upon perturbations, we subtracted the ATP5C1-Rosella data from the Rosella-LC3 data. Then, we assessed the proportion of general autophagy and mitophagy resources in basal and stressed conditions. After CCCP treatment, the frequency of general autophagic-vacuoles was reduced to 70% and mitophagy autolysosomes increased to 170% pointing that the autophagy machinery was shifted to mitochondria degradation (Fig. 4E). This observations suggest the redistribution of general autophagy resources between non-mitochondrial autophagy and mitophagy.

## Discussion

The autophagy field faces the challenge of identifying the different stages of autophagy and mitophagy in a high-throughput manner^5^. The steps and sub-cellular structures of autophagy and mitophagy are important for the proper interpretation of phenotypic traits. This compartmentalization cannot be represented with bulk population analysis techniques such as western blotting. Here, we demonstrate a platform that accounts for vesicular compartmentalization using unbiased automated analysis in combination with automated high-content imaging, providing high statistical power and assay sensitivity. In the present work, we developed a high-throughput and high-content phenotyping platform for autophagy and mitophagy. This platform enables not only the evaluation of autophagy and mitophagy homeostasis in basal conditions but also for recognizing changes due to stress conditions, showing its potential application for detecting disease specific fingerprints. Our results demonstrate the automated image analysis pipeline has similar performance to expert manual labeling in a test set of images. Mitochondrial counts detected by the image analysis algorithm tended to be higher than those detected by the human eye. This can be explained by the difference of Gaussians settings used which split the mitochondrial network when a reduction in cross-section is defined. This phenomena accounts for the necessary constriction preceding mitochondrial fission^24^. A difference of Gaussians uses the full dynamic range of graytones available, which results in an improved segmentation. In contrast, manual segmentation requires a computer screen that is unable to display the full dynamic range presenting a great challenge to segment small objects. The platform presented was evaluated with an assortment of compounds to test the modulation of autophagy and mitophagy. The patterns observed are in agreement with the expected results of each modulator’s mechanism of action. We observed that lysosome fusion blockers bafilomycin and chloroquine increased the proportion of autophagosomes and reduced the proportion of autolysosomes as previously reported^5^. First, we validated our workflow by comparing it to immunostaining analysis of total LC3-positive structures. As mentioned above, the count of LC3-positive structures is lower than the total vesicle count quantified by the reporter. This can be explained by the degradation of LC3 epitope after the acidification of vesicles. On the contrary, in the reporter line the RFP signal remains visible enabling the quantification of late autophagy pathway structures. We show that the vesicles quantified by immunostaining correspond mainly to pre-acidification structures, principally phagophores (Fig. 3B). Comparing the LC3-positive structures from immunostaining and phagophores from the autophagy reporter line validates that both systems present the same autophagy signature upon chloroquine induction (Fig. 3B).

Our measurements were conducted on monolayer cultures with optimal seeding and treatment windows that maximized the autophagy and mitophagy pathways response. In this framework, the mild starvation modulator EBSS did not induce significant alteration of autophagy structure proportion (Fig. 3C). This demonstrates that the effects observed with the chemical modulators correspond to *bona fide* pathway responses. This approach offers a population-based analysis to determine the probabilistic flux level from basal conditions to treatment conditions by accounting for the different subcellular structures of the pathway. Due to the substantial level of automation, this platform can be used for genetic and chemical screening and thereby accelerate current endeavors in precision medicine.

## Materials and Methods

### Human iPS cell culture and electroporation

Human iPS cell line A13777 (Gibco) derived with non-integrative methods was used. Cells were maintained in Essential-8 media (Thermo Fisher cat no. A1517001) in feeder free culture condition on laminin 521 (BioLamina) or Matrigel (BD). Cell passage and dissociation was performed with accutase (Thermo Fisher cat no. A11105-01). Cells were electroporated with a Lonza 4D nucleofector system (Lonza V4XP-3024) according to the manufacturer’s instructions. After passage or electroporation, cells were cultured with 10 µM Y27632 ROCK inhibitor (Sigma cat no. Y0503) for 24 h.

### Autophagy and mitophagy reporter system

The pH sensor fluorescent protein pHluorin (F64L, S65T, V193G and H231Q) was fused to DsRed and the mitochondrial or autophagosomal targeting sequence ATP5C1 or LC3II as previously described^10^. The coding sequence was introduced into the AAVS1 safe harbor locus as previously described^8,13^ using the targeting donor (Addgene plasmid # 22075) and TALE nucleases (Addgene plasmid #35432 and #35431).

### Pathway contribution dissection

Reporter lines were treated with an assortment of compounds to dissect the stages of mitophagy and autophagy. In order to achieve a homogeneous monolayer of cells in each well, the optimal post-seeding time and cell density were determined for the lines used. The optimal density was identified as 600 k/cm^2^ and the optimal post-seeding time as 8 hours, leaving a range of 40–60 cells per analyzed field. On 8 hours post-seeded cells, the minimal time required for assessing autophagy and mitophagy modulation were determined between 0.5 to 3 hours of treatment. The optimal imaging time of all compounds was identified as 3 hours after treatment, and was used for all experiments. Concentration gradients were tested to identify the minimal doses required to observe autophagy and mitophagy modulation without excessive cell toxicity after 3 hours of treatment, on 8 hours post-seeded cells. Final concentrations used from the ranges evaluated were 8 µM (8 µM–31.5 nM) bafilomycin A1 (Enzo); 8 µM (8 µM–31.5 nM) CCCP (Sigma cat no. C2759); 300 µM (300 µM–75 µM) chloroquine (Sigma cat no. C6628); 160 µM (160 µM–675 nM) DFP (Sigma cat no. D0879); 20 µM (20 µM–675 nM) oligomycin A (Sigma cat no. 75351); 160 µM (160 µM–675 nM) valinomycin (Sigma cat no. V3639); and 160 µM (160 µM–675 nM) rapamycin (Sigma cat no. R8781). Minimal laser-exposure time was optimized for the samples in basal conditions.

### Immunostaining

Cells were fixed on 4% PFA in PBS and permeabilized with PBS triton-X 0.2%. Total human LC3 monoclonal antibody (MBL cat no. M152-3) was incubated at dilution 1:500 overnight. Secondary antibody was goat anti-rabbit alexa fluor 647 (Thermo cat no. A32733) and used at dilution 1:1000.

### Lysosome quantification and nuclear contrast

Cells under basal conditions were treated with deep red lysotracker (Thermo cat no. L12492) at a dilution of 1:1000 for 30 minutes. For nuclear staining, cells were treated with 20 µM Hoechst 33342 for 10 minutes.

### Time-lapse live cell imaging

Culture dynamics and time lapse imaging was evaluated in a spinning disk CSU-X1 system (Zeiss) under controlled atmosphere conditions. Time-lapse imaging was performed for a single confocal plane. For three-dimensional pathway reconstruction, a single time point was evaluated. Reconstruction of 3D structures was performed with an Imaris (Bitplane) image processing 7.0 system.

### Microscopy for Rosella-LC3 and ATP5C1-Rosella

Confocal images were acquired on an Opera QEHS spinning disk microscope (Perkin Elmer) using a 60x water immersion objective (NA = 1.2). DsRed and pHluorin images were acquired in parallel using two cameras and binning 2. pHluorin was excited with a 488 nm laser and DsRed with a 561 nm laser. A 568 dichroic mirror was used to deviate the emitted light towards the corresponding cameras. pHluorin was detected on camera 1 behind a 520/35 bandpass filter and DsRed on camera 2 behind a 600/40 bandpass filter. For Rosella-LC3, five planes were set with 400 nm z-steps. For ATP5C1-Rosella, eleven planes were set with 400 nm z-steps. Scale of 1 pixel corresponds to 0.2152 µm in all the cases described.

### Image analysis for autophagy staging

First, the raw images (Fig. 2A–C, pHluorinImRaw and dsRedImRaw) were flatfield corrected on the basis of reference images from an adjustment plate. The flatfield corrected images were deconvolved using the deconvblind function (Fig. 2D,E, pHluorinDeconvolved and dsRedDeconvolved). The number of iterations was set to 10 and the initial estimate of the point spread function was generated with the PSFGenerator tool^17^. In the PSFGenerator, the Richards & Wolf 3D optical model was used using the parameters shown in Table S1. After deconvolution of the DsRed channel, differences of Gaussians were computed in order to highlight DsRed^pos^ vesicles via spatial bandpass filtering. For the detection of small DsRed^pos^ vesicles, a foreground image was computed via convolution with a Gaussian filter of size and standard deviation (GF-S-SD) 20 pixel and 1 pixel, respectively. The subtracted background image was returned from convolution with GF-S-SD 20 pixel and 7 pixel (Fig. 2F, dsRedDoG). The mask of small DsRed^pos^ vesicles was defined via thresholding (>400) (Fig. 2G, dsRedDoGmask). To further improve the sensitivity of detection for DsRed^pos^ vesicles, this approach was complemented with a top-hat filtering approach. Top-hat filtering of dsRedDeconvolved was done using the imtophat function and a disk shaped structuring element of radius 25 pixel (Fig. 2H, dsRedTopHat). Thresholding (>1200) returned the corresponding mask (Fig. 2I, dsRedTopHatMask). To refine dsRedTopHatMask, connected components with more than 500 pixels, overlapping with more than 10% of its pixels with the dsRedDoGmask, were substituted with the corresponding pixels in dsRedDoGmask (Fig. 2J, dsRedTopHatMaskSplit). Both, dsRedDoGmask and dsRedTopHatMaskSplit were combined using Boolean OR logic (Fig. 2K, dsRedMask1). To confirm the detection of DsRed^pos^ vesicles, a second difference of Gaussians was computed. Here the foreground image was convolved with a GF-S-SD of 11 pixel and 1 pixel while the background image was convolved with a GF-S-SD of 25 pixel and 6 pixel. The image resulting from subtraction of the background image from the foreground image (Fig. 2L, dsRedDoG2) was thresholded with gray tone value 1000 and objects were filtered by size between 200 and 2000 pixels (Fig. 2M, dsRedDoG2Mask). To split the vesicles, dsRedMask1 was used as a seed for a Euclidean distance transform and then the watershed function was applied. The resulting watershed mask (Fig. 2N, dsRedStencil1) was elementwise multiplied with the confirmative dsRedDoG2Mask (Fig. 2O, dsRedStencil2). The final DsRed^pos^ mask (Fig. 2P, dsRedMask) was computed via Boolean operation by pooling all pixels which were either present in dsRedMask1 or in dsRedStencil2. To add more sensitivity for the detection of non-acidic vesicles, segmentation steps based on the deconvolved pHluorin channel were added. First, a difference of Gaussians was computed to segment larger vesicles. The GF-S-SD used to compute the foreground image was 100 pixel and 1 pixel, and the subtracted background image was convolved with a GF-S-SD of 100 pixel and 5 pixel (Fig. 2Q, GreenDoG). The mask of big green fluorescent vesicles was returned from intensity thresholding (>1000) (Fig. 2R, GreenDoGMask). To detect edges in the deconvolved pHluorin channel, a Laplacian of Gaussian filter with size 20 pixel and standard deviation 1 pixel was applied (Fig. 2S, GreenLoG), and pixels with values < −2000 were returned into the mask of edges (Fig. 2T, GreenLoGmask). The mask combining all vesicles detected in the pHluorin channel was computed via Boolean OR operation where the mask of big green fluorescent vesicles and the mask of edges were merged and connected components with less than 10 pixels were removed (Fig. 2U, pHluorinMask). Ratio images (Fig. 2V, RatioIm) were computed by applying convolution GF-S-SD of 5 pixel and 2 pixel to the raw pHluorin and the raw DsRed channel. The ratio image was computed by elementwise division between the blurred pHluorin image and the blurred DsRed image. To detect potentially missed autolysosomes, RatioIm was complemented using the imcomplement function (Fig. 2W, RatioImComp), and top-hat filtered using a disk-shaped structuring element of radius 15 pixel. Connected components above threshold 1.5 (Fig. 2X, AutoLysoMaskCandidates) were further validated as autolysomes by comparing their green fluorescence with the green fluorescence in the neighborhood defined by dilatation with a disk shaped structuring element of radius 7 pixel. Only when the neighborhood was at least 50% brighter than the vesicle and the vesicle volume was larger than 100 pixel, it was retained as an autolysosome candidate (Fig. 2Y, AutoLysoMask). To detect autophagosomes based on their hollow vesicle property, an algorithm combining Fourier transform and Euler filtering was implemented. First, the GreenDoG image was plane by plane filtered with a Butterworth high pass filter in the Fourier transform domain. The cutoff frequency of the Butterworth filter was set to 10 and the order of the filter was set to 5. The resulting image (Fig. 2Z, GreenDoGFTB) was binarized via thresholding (>150) (Fig. 2AA, GreenDoG150). The resulting mask was maximum projected along the z axis. Objects with less than 20 pixel were removed, and median filtering using a 3 × 3 structuring element was applied. To prepare for the downstream analysis of connected components, the mask was further opened with a disk shaped structuring element of radius 1 pixel (Fig. 2AB, GreenDoG150b). Euler numbers were used to detect connected components containing holes. Indeed, an Euler number of 0 indicates that a connected component contains exactly one hole. Only connected components with Euler number 0 were retained (Fig. 2AC, EulerZero). For further filter refinement, the proportions between object and hole size were evaluated. For that purpose the imfill function was used in order to create a filled mask (Fig. 2AD, EulerZeroFilled). In the proportion filter, only objects with a ratio between area and filled area larger than 1.01, and a difference between filled area and area larger than 20 were retained (Fig. 2AE, EulerSelect). To segment the holes, the mask was inverted and the background was removed by applying a size threshold (10000 pixels) (Fig. 2AF, EulerZeroHoles). Next, the imreconstruct function was used, with the hole mask described above as the seed mask, and the FilledMask as the limiting mask. Finally, the shapes of autophagosome candidate vesicles were restored via morphological operations by applying image opening with a disk shaped structuring element of radius 5 pixel. For 3D reconstruction, only those planes which were already positive in GreenDoG150 were retained (Fig. 2AG, EulerMask). To detect remaining autophagosomes, Hough transforms for circle detection were applied to the raw pHluorin channel. To minimize false positive detections, the already identified vesicle pixels were substituted by low pass filtered pixels. To highlight the not yet detected circular vesicles, graytone erosion with a disk shaped structuring element of radius 2 pixel was applied (Fig. 2AH, HoughInput). For the detection of circles, the function imfindcircles was used, where the radius was set to a range between 3–30 pixel. To remove false positives from the Hough transform algorithm, each candidate circle was further analyzed with respect to its value in the raw green and red channels. Only candidate circles with a large median absolute deviation among raw pHluorin pixels (>20), and a low 0.9th quantile of raw DsRed^pos^ pixels (<300) were retained (Fig. 2AI, HoughOutput). The mask of all autophagosome candidates was computed by combining autophagosome candidates detected via the Fourrier-Euler algorithm or the Hough algorithm. To minimize the number of false positive autophagosomes, filters based on size, shape, and the ratio between vesicle border and vesicle center intensities were applied. The allowed sizes were set to the range between 50–10,000 pixel (Fig. 2AJ, AutophagosomeCandidates). For shape evaluation, the vesicle surface was defined by erosion with a sphere shaped structuring element of radius 1 pixel. Two sphericity indices were computed as shown in Fig. 2 (Fig. 2AO). Only vesicles with SphericityIdx1 > 1 and SphericityIdx2 > 1.5 were retained. For classification, all vesicles detected via the different approaches shown above were combined via Boolean operations. In order to maintain vesicle splitting, the perimeter of the autophagosome mask was excluded from the pooled vesicle mask. To exclude DsRed^neg^ vesicles from the downstream analysis, the mean intensity in the raw DsRed channel was measured for all connected components. Only vesicles with a mean intensity above 300 and not touching the border of the image were considered for the classification (Fig. 2AK, VesiclesAll). For the remaining vesicles, the eccentricity was computed and all connected components with eccentricity >0.9 were collected in the mask named NonCircularVesicleMask (Fig. 2AL). The classification of the segmented vesicles was designed to classify four vesicle types, namely phagophores, autophagosomes, early autolysosomes, and late autolysosomes. For this purpose, a progressive exclusion algorithm was implemented. First, vesicles overlapping with the autophagosome mask and not overlapping with the NonCircularVesicleMask were classified as autophagosomes. Three condition sets were defined to classify the remaining vesicles as phagophores. Case1: vesicles with at least 25% overlap with both the pHluorinMask and the DsRedMask, a median ratio above 2 and without overlap with the AutoLysoMask. Case2: the 3rd quantile of the green channel was above 7500 and the 3rd quantile of the red channel above 4000. Case3: the green fluorescence in the center of the vesicle was at least 25% brighter than at the vesicle’s surface. For the remaining vesicles that were not classified as autophagosomes or phagophores, two conditions were defined for the vesicle labeling as late autolysosome. Case1: vesicles with at least 25% overlap with the DsRedMask and less than 10% overlap with the pHluorinMask or, alternatively, a median ratio below or equal to 2. Case2: the green fluorescence was lower in the vesicle center than at the surface. Remaining vesicles, not classified as autophagosomes, phagophores, or late autolysosomes were defined as early autolysosomes if they had at least 25% overlap with the DsRedMask and less or equal to 25% overlap with the pHluorinMask, or if they overlapped with the AutoLysoMask. Resulting segmented structures are shown for DsRed and pHluorin (Fig. 2AM–AN).

### Image analysis for mitophagy staging

For the segmentation of mitochondria, the DsRed channel foreground signal was computed via convolution GF-S-SD of 50 pixel and 1 pixel (Fig. 4B). The subtracted background signal was computed via convolution with a GF-S-SD of 50 pixel and 2 pixel. Pixels with graytone values above 12 in this difference of Gaussians image (Fig. 4B, MitoDoG) were defined as mitochondrial pixels (Fig. 4B, MitoMask). MitophagyEvents were defined via a combination of green to red fluorescence ratio analysis and morphological filtering based on difference of Gaussians thresholding. First, 26 connected components within MitoMask were defined as mitophagy event markers (Fig. 4B, MitophagySeedMask) if the mean ratio value within the connected components was below 0.6. To refine the shape of the detected mitophagy events, the imreconstruct function was used with the parameters MitophagySeedMask and MitophagyLimitingMask (Fig. 4B). The MitophagyLimitingMask was defined by pixel values above 50 in a difference of Gaussians of the DsRed channel (Fig. 4B, MitophagyDoG). MitophagyDoG was defined by GF-S-SD of 50 pixel and 1 pixel for the foreground and GF-S-SD of 50 pixel and 5 pixel for the background.

### Microscopy for the lysotracker assay

Images were acquired on an Opera QEHS High content screening microscope using a 60x water immersion objective (NA = 1.2). Lysotracker deep red was excited with a 640 nm laser and detected with a 690/70 bandpass filter using camera binning 2. Z-stacks were defined to contain 11 planes with 400 nm z-steps.

### Image analysis for the lysotracker assay

Deconvolution of raw images (Fig. S1A, LysoTDR) was done as described above according to the settings shown in Supplementary Table 1 (Fig. S1B, LysoTDR_deconvolved and Table S1). For the segmented lysotracker positive vesicles, an algorithm with different morphological filters was implemented. First, a difference of Gaussians was computed using a GF-S-SD of 100 pixel and 1 pixel for convolving the foreground image and a GF-S-SD of 100 pixel and 5 pixel for convolving the subtracted background image (Fig. S1C, LysoTDR_DoG). A first mask with Lysotracker pixels was returned by thresholding (>2000) LysoTDR_DoG (Fig. S1E, LysoTracker_DoG_Mask). An additional detection option was implemented using convolution of LysoTDR_deconvolved with a Laplacian of Gaussian of GF-S-SD 20 pixel and 1 pixel (Fig. S1D, LysoTDR_LoG). The second mask of lysotracker pixels was returned by retaining pixels with graytone values < −2000 in LysoTDR_LoG (Fig. S1F, LysoTDR_LoG_Mask). The final mask of lysotracker stained vesicles was computed via Boolean OR combination of both masks and size exclusion of connected components with less than 10 pixels (Fig. S1G, LysoTDR_Mask). To compute the major axis length of each vesicle, LysoTDR_Mask was maximum projected and subsequently the function regionprops was used to extract the major axis length of each connected component in the projected mask.

## Supplementary information

Supplemenrary Information

Supplemenrary Video 1

Supplemenrary Video 2

Supplemenrary Video 3

Supplemenrary Video 4

Supplemenrary Video 5

Supplemenrary Video 6

Supplemenrary Video 7
