# Supplementary material for: Automated high-throughput high-content autophagy and mitophagy analysis platform
Source: Sci Rep. 2019 Jul 1;9:9455. doi: 10.1038/s41598-019-45917-2 (PMC6603000; doi:10.1038/s41598-019-45917-2)
Supplement: Supplementary file 1 — Supplemenrary Information [file 41598_2019_45917_MOESM1_ESM.docx]

**Supplementary Information**

**Automated high-throughput high-content autophagy and mitophagy analysis platform**

Jonathan Arias-Fuenzalida^1,2,5,6^, Javier Jarazo^1,2,6^, Jonas Walter^1,2,6^, Gemma Gomez-Giro^1,2,4^, Julia I. Forster^1,3^, Rejko Krueger^1,3^, Paul M.A. Antony^1,3,7^ & Jens C. Schwamborn^1,2,7,8^

1 Luxembourg Centre for Systems Biomedicine (LCSB), University of Luxembourg, Luxembourg, 7 avenue des Hauts-Fourneaux

2 Laboratory of Developmental and Cellular Biology

3 Laboratory of Experimental Neurobiology

4 Max Planck Institute for Molecular Biomedicine, Laboratory of Cell and Developmental Biology, Roentgenstrasse 20, Muenster, Germany

5 Graduate School of Biostudies, Kyoto University, Kyoto 606-8501, Japan

6 Authors equally contributed to this article

7 Correspondence should be addressed to P.A. (paul.antony@uni.lu) and J.S. (jens.schwamborn@uni.lu)

**Titles and legends to Supplementary Figures**

**Fig. S1.** Image analysis workflow for the Lysotracker assay in reporter lines. The names of the images match those in the online methods. (**A**) Raw images for lysotracker, LysoTDR. (**B**) LysoTDR_deconvolved. (**C**) LysoTDR_DoG. (**D**) LysoTDR_LoG. (**E**) LysoTracker_DoG_Mask. (**F**) LysoTDR_LoG_Mask. (**G**) LysoTDR_Mask. Projected major axis length of each connected component is represented. Scale bars indicate 20 µm and 3x zoomed insets are highlighted with yellow boxes.

**Video S1.** Autophagy dynamics in iPS cells. The Rosella-LC3 healthy-control line was imaged for 1 hour 15 min. Arrows depict examples of phagophores and autophagic vacuoles. Squares represent the close-up area of Video S2 and Video S3. Phagophores mobilized throughout the cell. Autophagic vacuoles appear and interact with other vesicles. Speed 25 fps. Scale bar, 20 µm.

**Video S2.** Autophagy dynamics in iPS cells. The Rosella-LC3 healthy-control line was imaged for 1 hour 15 min. Close-up of Video S1 shows the emergence of autophagosomes of dsRED^pos^pHluorin^pos^ and of autolysosomes of dsRED^pos^pHluorin^neg^ lumen. Speed 25 fps. Scale bar, 5 µm.

**Video S3.** Autophagy dynamics in iPS cells. The Rosella-LC3 healthy-control line was imaged for 1 hour 15 min. Close-up of Video S1 shows the partitioning of autophagic vacuoles previous to telophase. Speed 25 fps. Scale bar, 5 µm.

**Video S4.**  Autophagy dynamics in iPS cells. Navigation through a 3D reconstruction of the Rosella-LC3 healthy-control culture. Phagophores, autophagosomes, and late autolysosomes are indicated. The fluorescence intensity of the pH sensor highlights the transitions in the autophagy cycle. Dynamic scale bar.

**Video S5.** Mitophagy dynamics in iPS cells. The ATP5C1-Rosella healthy-control line was imaged for 1 hour 15 min. Mitochondrial networks dynamically reorganize and split. Autolysosomes that are responsible for mitophagy are visible. Speed 25 fps. Scale bar, 20 µm.

**Video S6.** Mitophagy dynamics in iPS cells. The ATP5C1-Rosella healthy-control line was imaged for 1 hour 15min. Close-up of video S5. Autolysosomes associate with the mitochondrial network and present a “kiss and run” monitoring behavior. Mitochondrial networks dynamically reorganize and split. Speed 25fps. Scale bar 20 µm.

**Video S7.** Mitophagy dynamics in iPS cells. The ATP5C1-Rosella healthy-control line was imaged for 1 hour 15min. Close-up of video S5. Autolysosomes associate with the mitochondrial network and present a “kiss and run” monitoring behavior. Speed 25fps. Scale bar 20 µm.

**Table S1**. PSF generator settings

| Channel | pHluorin | DsRed | LysoTDR |
| --- | --- | --- | --- |
| Refractive index immersion | 1.3 | 1.3 | 1.3 |
| Accuracy computation | Best | Best | Best |
| Wavelength | 520 nm | 600 nm | 690 |
| NA | 1.2 | 1.2 | 1.2 |
| Pixel size | 215.2 nm | 215.2 nm | 215.2 nm |
| Z-step | 400 nm | 400 nm | 400 nm |
| Size XYZ | 256, 256, 5 | 256, 256, 5 | 256, 256, 11 |
